# Genetic Determinants of UV-Susceptibility in Non-Melanoma Skin Cancer

**DOI:** 10.1371/journal.pone.0020019

**Published:** 2011-07-08

**Authors:** Marleen M. Welsh, Margaret R. Karagas, Jacquelyn K. Kuriger, Andres Houseman, Steven K. Spencer, Ann E. Perry, Heather H. Nelson

**Affiliations:** 1 Department of Preventive Medicine and Biometrics, Uniformed Services University of the Health Sciences, Bethesda, Maryland, United States of America; 2 Department of Community and Family Medicine, Dartmouth Medical School, Lebanon, New Hampshire, United States of America; 3 Norris Cotton Cancer Center, Dartmouth Medical School, Lebanon, New Hampshire, United States of America; 4 Masonic Cancer Center, University of Minnesota, Minneapolis, Minnesota, United States of America; 5 Division of Epidemiology and Community Health, University of Minnesota, Minneapolis, Minnesota, United States of America; 6 Department of Community Health, Brown University, Providence, Rhode Island, United States of America; 7 Center for Environmental Health and Technology, Brown University, Providence, Rhode Island, United States of America; 8 Department of Medicine, Dartmouth Hitchcock Medical Center, Lebanon, New Hampshire, United States of America; 9 Department of Pathology, Dartmouth Hitchcock Medical Center, Lebanon, New Hampshire, United States of America; The University of Queensland, Australia

## Abstract

A milieu of cytokines and signaling molecules are involved in the induction of UV-induced immune suppression and thus the etiology of non-melanoma skin cancer (NMSC). Targeting the UV-induced immunosuppression pathway, and using a large population based study of NMSC, we have investigated the risk associated with functional variants in 10 genes (*IL10*, *IL4*, *IL4R*, *TNF*, *TNFR2*, *HTR2A*, *HRH2*, *IL12B*, *PTGS2*, and *HAL*). The most prominent single genetic effect was observed for *IL10*. There was increasing risk for both basal cell carcinoma (BCC) and squamous cell carcinoma (SCC) with increasing number of variant *IL10* haplotypes (BCC: p_trend_ = 0.0048; SCC: p_trend_ = 0.031). Having two *IL10* GC haplotypes was associated with increased odds ratios of BCC and SCC (OR_BCC_ = 1.5, 95% CI 1.1–1.9; OR_SCC_ = 1.4, 95% CI 1.0–1.9), and these associations were largely confined to women (OR_BCC_ = 2.2, 95% CI 1.4–3.4; SCC: OR_SCC_ = 1.8, 95% CI 1.1–3.0). To examine how combinations of these variants contribute to risk of BCC and SCC, we used multifactor dimensionality reduction (MDR) and classification and regression trees (CART). Results from both of these methods found that in men, a combination of skin type, burns, *IL10*, *IL4R*, and possibly *TNFR2* were important in both BCC and SCC. In women, skin type, burns, and *IL10* were the most critical risk factors in SCC, with risk of BCC involving these same factors plus genetic variants in *HTR2A*, *IL12B* and *IL4R*. These data suggest differential genetic susceptibility to UV-induced immune suppression and skin cancer risk by gender.

## Introduction

While difficult to measure precisely due to lack of inclusion in national cancer registries, the incidence of non-melanoma skin cancers (NMSCs) in the US is estimated to have matched that of all other cancers combined in 2005 [1] and has been increasing dramatically over the last 25 years [2], with greater increases in women than in men. While predisposing risk factors for NMSC include pigmentation, gender, arsenic or ionizing radiation exposure, and genetic disorders such as Xeroderma pigmentosum and basal cell nevus syndrome [3], exposure to ultraviolet radiation (UVR) is arguably the most important to the development of NMSC.

Separate from mutagenic effects, this type of UV is also implicated in the etiology of cancer via immunosuppression. This immunomodulation results in depletion of the Langerhans cells from the epidermis, improper antigen presentation in the lymph nodes, a shift towards Th2 responses, and development of tumor antigen-specific T regulatory cells, resulting in blocked immune surveillance and tumor outgrowth [4], [5], [6], [7], [8], [9]. Studies using antibody-mediated cytokine suppression and genetically engineered mice have found a wealth of downstream mediators that are critical in UV-induced immune responses.

Strains of inbred mice may be classified into distinct categories of UV-susceptibility and UV-resistance following equal doses of UVR, implicating a genetic component to susceptibility to UV-induced immunosuppression [10]. In New Zealand black (NZB) mice, a strain that exhibits deficiencies in maintaining self-tolerance which lead to a murine form of human systemic lupus erythematosis [11], there was a seven fold increase in sensitivity to UVB in females compared to males, implicating possible gender differences in this process. In both sexes, UV-susceptible mice have higher incidence rates of skin cancer than UV-resistant mice [12]. Preliminary data from two small studies in humans suggest a similar association. An estimated 35–40% of humans may be classified as having a UV-susceptible phenotype, as measured by a contact hypersensitivity test [13], [14]. One of these studies also found that over 90% of skin cancer patients were of the UV-susceptible phenotype [13], further linking cancer development, genetic susceptibility, and UV immunosuppression.

In this study, we analyzed purported functional variants in 10 key mediators of UV-induced immunosuppression and risk of NMSC, including investigation of both single and multiple gene effects. Additionally, we investigated whether these effects varied by gender, as past studies have indicated possible gender differences in risk of NMSC from UV-induced immunosuppression.

## Methods

### Study Population

Newly diagnosed cases of histologically confirmed BCC and SCC in New Hampshire were identified using an incident survey established through the collaboration of dermatologists, dermatopathologists, and pathology laboratories throughout the state and bordering regions from July 1, 1993 to June 30, 1995 (series 1) and July 1, 1997 to March 30, 2000 (series 2) [2]. The study design of The New Hampshire Health Study has been described previously by Karagas et *al*. [2]. Briefly, eligibility criteria for cases were as follows: 1) between 25 and 74 years of age, 2) had a listed telephone number, and 3) spoke English. All eligible invasive SCC cases and a ratio of approximately two to one BCC cases in series 1 and one to one ratio in series 2 were selected to take part in the study. The BCC cases were randomly sampled in order to ensure representativeness of age, sex, and anatomic site for all incident BCCs within New Hampshire. Controls aged 25–64 years were identified from the New Hampshire State Department of Transportation files and those aged 65–74 years were obtained from enrollment lists from the Center for Medicaid and Medicare Services. Potential controls were frequency-matched on age and gender to the combined distribution of case groups.

A personal interview was conducted with consenting cases and controls, with about 80% of cases and 72% of controls agreeing to participate. The interviews, usually conducted in the participant's home, covered demographic factors, pigmentation characteristics, sun exposure and sensitivity, and other factors [2]. Blood draws and/or buccal samples were obtained from cases and controls from both phases of the study for DNA analysis. Approximately 85% of subjects consented to a providing a DNA sample; among those that consented, 90% provided a blood-derived sample. All study protocol and materials were approved by the Dartmouth College Committee for the Protection of Human Subjects, and all participants provided informed consent.

### Genotyping

Functional polymorphisms in 10 immune-related genes were genotyped (Table 1). DNA was extracted from peripheral blood lymphocytes of consented cases and controls using Qiagen Genomic DNA extraction kits (Valencia, CA). For quality assurance purposes, 10% of blood and buccal samples were used as integrated duplicates, to which researchers were blinded at the time of genotyping. The genotypes of the embedded duplicates were compared afterwards to that of the original samples to calculate the genotyping error rate. All genotype error rates were 5% or less.

**Table 1:** Functional polymorphisms in the UV-induced immunosuppression pathway.

Gene name (HUGO)	Chromosome	dbSNP ID	Polymorphism	Functionality
*HAL*	12q22-24.1	rs7297245	A→G, codon 439, exon 16	n/a
*HTR2A*	13q14-21	rs6311	T→C, −1438, promoter	T>C expression [15]
*HRH2*	5q35.2	rs1800689	G→A, codon 181, exon 1	n/a
*TNF*	6p21.3	rs1800629	G→A, −308, promoter	A>G expression[16], [17]
*TNFR2*	1p36.2	rs1061622	T→G, codon 196, exon 7	G(R)>T(M), NF-kB activation [18]
*IL12B*	5q31.1-33.1	rs3212227	A→C, +16974, 3′UTR	A>C IL-12 p70 secretion [19]
*IL10*	1q31-32	rs1800896	G→A, −1082, promoter	GCC>ATA expression [20]
		rs1800871	C→T, −819, promoter	
		rs1800872	C→A, −592, promoter	
*PTGS2*	1q25.2-25.3	rs689466	G→A, −1195, promoter	A>G expression [21]
*IL4*	5q31.1	rs2243250	C→T, −590, promoter	T>C expression [22]
*IL4R*	16p11.2-12.	rs1805010	A→G, codon 75, exon 5	GG(VR)>AA (IQ) sensitivity to IL-4 [23], [24], [25]
		rs1801275	A→G, codon 576, exon 11	

Genotyping for the histidase I439V SNP (rs7297245) was performed by PCR-RFLP analysis. A 333 base pair fragment of exon 16 of the *HAL* locus was amplified using primers as previously described [26], [27]. Genotyping of four SNPs (rs1800629, rs3212227, rs2243250, rs1805010) was performed on the Taqman allelic discrimination platform (Applied Biosystems, Foster City, CA). Taqman primers, probes, and conditions are available upon request. All other SNPs (rs6311, rs1800689, rs1061622, rs1800896, rs1800871, rs1800872, rs689466, rs1801275) were genotyping by the University of Minnesota Biomedical Genomics Center using the Sequenom platform. Two *IL10* SNPs, rs1800871 and rs1800872, were in high linkage disequilibrium (D' = 0.99) in our population, and therefore rs1800872 was not included in the analysis.

### Logistic Regression

Adjusted odds ratios (ORs) and 95% confidence intervals for each SNP were obtained using unconditional logistic regression adjusting for age at diagnosis (continuous), gender, skin type and lifetime number of severe sunburns. Self-reported sunburn history was described as the lifetime number of painful sunburns that last 2 or more days. For all analyses, cutoffs were determined based on the distribution in controls, creating groups of no (0), low (1–3), and high (≥4) burns. For stratified analysis, the no and low groups were combined, as done in previous analysis [27]. The measure of skin type, skin's reaction to acute sun exposure, was also trichotomized. Responses were grouped as tan, burn then tan, or burn, if they responded that they burned and peeled or burned and freckled.

### Multifactor Dimensionality Reduction

Multifactor dimensionality reduction (MDR) is a nonparametric analysis method in which multiple variables, or attributes, are reduced into a single, binary attribute with “low risk” and “high risk” categories. The specifics of the algorithm used have been previously described [28]. Analysis was conducted for 1 through 5 factor attribute combinations. Ten-fold cross validation was used to develop training and testing sets for each analysis. For those models where the cross validation consistency (CVC) was not 10/10, the true testing balance accuracy (TBA) of that combination was recalculated using only those variables from the most accurate model. This TBA and the original CVC were reported. To determine statistical significance, a normal distribution of testing balance accuracy values was derived by permutating the data 1000 times. As in logistic regression, all genotypes were binary except *IL10* and *IL4R*. Age was dichotomized as 60 and younger or over 60 years. All other variables were coded as described above.

### Classification and Regression Trees

Classification and regression trees (CART) is a decision tree-based analysis method that uses binary recursive partitioning to create prediction rules for the outcome of interest. The details of the algorithm used have been previously described [29], [30]. All variables were coded the same as in MDR analysis. A 10-fold cross validation was used for model building, and the optimal tree was chosen as the one with the highest ROC value. To compute the prediction rate, a bootstrap aggregation method was applied to the data, in which 500 trees were averaged to create a “committee of experts” model to best explain the variability in the data.

## Results

Evaluation of genetic variants was conducted in 2493 participants (852 controls, 931 BCC, 710 SCC). The frequency distributions for age, sex, and other known risk factors for NMSC, such as skin type and lifetime number of severe sunburns, are shown in Table 2. The average age of participants was 61.3 years (SD = 10.6) for controls, 58.7 years (SD = 11.1) for BCC, and 64.1 years (SD = 8.7) for SCC. In all groups, there were more men than women. BCC and SCC cases were more likely to have experienced severe sunburns and were more likely to burn than controls.

**Table 2:** Population Demographics and Exposures.

	Controls N = 852	BCC N = 931	SCC N = 710
**Age (SD)**	61.3 (10.6)	58.7 (11.1)	64.1 (8.7)
**Gender**			
Male	510 (59.9%)	521 (56.0%)	451 (63.5%)
Female	342 (40.1%)	410 (44.0%)	259 (36.5%)
**Skin reaction to 1^st^ strong summer sun for one hour**			
Tan	162 (19.1%)	72 (7.7%)	72 (10.2%)
Burn then tan	426 (50.2%)	458 (49.3%)	321 (45.3%)
Burn	261 (30.7%)	400 (43.0%)	316 (44.6%)
missing	3	1	1
**Lifetime severe sunburns**			
Low (0–3)	525 (65.2%)	459 (50.9%)	346 (50.7%)
High (≥4)	280 (38.8%)	442 (61.2%)	336 (49.3%)
missing	47	30	28

Unconditional logistic regression was used to examine the individual effects of each SNP or haplotype in the pathway. While controlling for age, sex, skin type, and lifetime number of sunburns, *IL10* GC haplotypes were associated with increased risk of both BCC (Table 3) and SCC (Table 4). Having two GC haploytypes, which is associated with higher *IL10* expression than the AT allele [20], was associated with increased ORs of BCC and SCC (OR_BCC_ = 1.5, 95% CI 1.1–1.9; OR_SCC_ = 1.4, 95% CI 1.0–1.9), although the association with SCC was of borderline significance. There was evidence of a gene dosage effect, with increasing ORs associated with number of GC haplotypes (BCC: p_trend_ = 0.0048; SCC: p_trend_ = 0.031). After using the conservative Bonferroni multiple comparisons correction for 10 independent test (p<0.005), the trend in BCC remains significant, but the trend is no longer significant in SCC. When the number of *IL10* GC haplotypes was treated as a continuous variable, there was a significant 20% increase in risk of both BCC and SCC associated with each additional GC haplotype (BCC: OR_BCC_ = 1.2, 95% CI 1.1–1.4; SCC: OR_SCC_ = 1.2, 95% CI 1.0–1.4), although again this association was of borderline significance in SCC. As shown in a previous study in this population, there was no significant association between *HAL* genotype and BCC or SCC [27].

**Table 3:** Logistic regression analysis of individual SNPs or haplotypes and BCC.

BCC	Controls N(%)	Cases N (%)	OR (95% CI)^a^	Men	Women
*HAL*					
AA	511 (61.2)	541 (59.8)	Referent	Referent	Referent
AG	281 (33.7)	309 (34.1)	1.0 (0.8–1.3)	0.9 (0.7–1.2)	1.2 (0.8–1.6)
GG	43 (5.2)	55 (6.1)	1.2 (0.8–1.8)	1.5 (0.8–2.8)	0.9 (0.5–1.7)
AG/GG	324 (38.8)	364 (40.2)	1.1 (0.9–1.3)	1.0 (0.8–1.3)	1.1 (0.8–1.5)
*HTR2A*					
CC	267 (33.1)	323 (36.3)	Referent	Referent	Referent
CT	407 (50.5)	406 (45.7)	0.9 (0.7–1.1)	0.9 (0.7–1.2)	0.8 (0.6–1.2)
TT	132 (16.4)	160 (18.0)	1.0 (0.8–1.4)	0.9 (0.6–1.4)	1.1 (0.7–1.8)
*HRH2*					
GG	714 (86.8)	791 (88.8)	Referent	Referent	Referent
AG/AA	109 (13.2)	100 (11.2)	0.8 (0.6–1.1)	0.8 (0.5–1.1)	0.9 (0.6–1.4)
*TNF*					
GG	571 (70.0)	612 (68.5)	Referent	Referent	Referent
GA	223 (27.3)	265 (29.6)	1.1 (0.9–1.4)	1.1 (0.9–1.5)	1.1 (0.8–1.5)
AA	22 (2.7)	17 (1.9)	0.8 (0.4–1.5)	0.5 (0.2–1.4)	1.2 (0.4–3.1)
GA/AA	245 (30.0)	282 (31.5)	1.1 (0.9–1.3)	1.1 (0.8–1.4)	1.1 (0.8–1.5)
*TNFR2*					
TT	503 (61.6)	558 (62.9)	Referent	Referent	Referent
GT	268 (32.8)	283 (31.9)	1.0 (0.8–1.2)	0.9 (0.7–1.2)	1.1 (0.8–1.5)
GG	45 (5.5)	46 (5.2)	0.9 (0.6–1.5)	0.7 (0.4–1.2)	1.7 (0.8–3.9)
GT/GG	313 (38.4)	329 (37.1)	1.0 (0.8–1.2)	0.9 (0.7–1.1)	1.1 (0.8–1.5)
*IL12B*					
AA	500 (65.5)	555 (65.7)	Referent	Referent	Referent
AC	230 (30.1)	250 (29.6)	1.0 (0.8–1.2)	0.9 (0.6–1.2)	1.1 (0.8–1.5)
CC	33 (4.3)	40 (4.7)	1.0 (0.6–1.7)	1.3 (0.6–2.5)	0.8 (0.4–1.7)
AC/CC	263 (34.5)	290 (34.3)	1.0 (0.8–1.2)	0.9 (0.7–1.2)	1.1 (0.8–1.5)
*IL10* G-C alleles					
0	243 (29.6)	236 (26.2%)	Referent	Referent	Referent
1	393 (47.8)	413 (45.8%)	1.1 (0.9–1.4)	1.0 (0.7–1.3)	1.2 (0.9–1.8)
2	186 (22.6)	252 (28.0%)	1.5 (1.1–1.9)	1.1 (0.8–1.6)	2.2 (1.4–3.4)
Test for trend			p = 0.0048	p = 0.45	p = 0.0005
*PTGS2*					
GG	32 (3.8)	26 (2.9)	Referent	Referent	Referent
AG	260 (31.1)	296 (32.7)	1.4 (0.8–2.4)	1.1 (0.6–2.1)	2.3 (0.8–6.6)
AA	544 (65.1)	582 (64.4)	1.2 (0.7–2.2)	1.1 (0.6–2.0)	1.9 (0.7–5.6)
GG/AG	292 (34.9)	322 (35.6)	Referent	Referent	Referent
AA	544 (65.1)	582 (64.4)	1.2 (0.7–2.2)	1.1 (0.6–2.0)	1.9 (0.7–5.6)
*IL4*					
CC	608 (75.9)	675 (75.7)	Referent	Referent	Referent
CT	174 (21.7)	197 (22.1)	1.0 (0.8–1.3)	0.9 (0.7–1.3)	1.1 (0.7–1.6)
TT	19 (2.4)	20 (2.2)	0.9 (0.5–1.8)	0.7 (0.3–1.4)	2.7 (0.7–10.5)
CT/TT	193 (24.1)	217 (24.3)	1.0 (0.8–1.2)	0.9 (0.7–1.2)	1.1 (0.8–1.6)
*IL4R* var alleles					
0	170 (23.1)	146 (17.9)	Referent	Referent	Referent
1–2	481 (65.4)	576 (70.8)	1.4 (1.1–1.8)	1.4 (1.0–1.9)	1.5 (1.0–2.3)
3–4	84 (11.4)	92 (11.3)	1.3 (0.9–1.9)	1.3 (0.8–2.1)	1.3 (0.7–2.3)
Test for trend			p = 0.073	p = 0.14	p = 0.31

a ORs adjusted for gender, age at tumor diagnosis, skin reaction to acute sun exposure, and lifetime number of severe sunburns.

**Table 4:** Logistic regression analysis of individual SNPs or haplotypes and SCC.

SCC	Controls N(%)	Cases N (%)	OR (95% CI)^a^	Men	Women^a^
*HAL*					
AA	511 (61.2)	410 (58.9)	Referent	Referent	Referent
AG	281 (33.7)	243 (34.9)	1.1 (0.9–1.3)	0.9 (0.7–1.2)	1.5 (1.0–2.2)
GG	43 (5.2)	43 (6.2)	1.3 (0.8–2.0)	1.3 (0.7–2.4)	1.3 (0.7–2.7)
AG/GG	324 (38.8)	286 (41.1)	1.1 (0.9–1.4)	0.9 (0.7–1.2)	1.5 (1.0–2.1)
*HTR2A*					
CC	267 (33.1)	223 (33.4)	Referent	Referent	Referent
CT	407 (50.5)	350 (52.4)	1.0 (0.8–1.3)	0.9 (0.7–1.2)	1.2 (0.8–1.8)
TT	132 (16.4)	95 (14.2)	0.8 (0.6–1.2)	0.8 (0.5–1.2)	0.9 (0.5–1.6)
*HRH2*					
GG	714 (86.8)	587 (86.6)	Referent	Referent	Referent
AG/AA	109 (13.2)	91 (13.4)	1.1 (0.8–1.4)	1.0 (0.6–1.4)	1.3 (0.8–2.2)
*TNF*					
GG	571 (70.0)	476 (69.9)	Referent	Referent	Referent
GA	223 (27.3)	188 (27.6)	1.0 (0.8–1.2)	1.1 (0.8–1.5)	0.8 (0.5–1.2)
AA	22 (2.7)	17 (2.5)	0.9 (0.5–1.8)	0.7 (0.3–1.7)	1.4 (0.5–4.1)
GA/AA	245 (30.0)	205 (30.1)	1.0 (0.8–1.2)	1.1 (0.8–1.4)	0.8 (0.6–1.2)
*TNFR2*					
TT	503 (61.6)	396 (57.6)	Referent	Referent	Referent
GT	268 (32.8)	259 (37.7)	1.2 (1.0–1.5)	1.3 (0.9–1.7)	1.1 (0.8–1.6)
GG	45 (5.5)	33 (4.8)	0.9 (0.6–1.5)	0.8 (0.4–1.4)	1.5 (0.6–3.7)
GT/GG	313 (38.4)	292 (42.4)	1.2 (0.9–1.4)	1.2 (0.9–1.5)	1.1 (0.8–1.6)
*IL12B*					
AA	500 (65.5)	416 (65.2)	Referent	Referent	Referent
AC	230 (30.1)	196 (30.7)	1.1 (0.9–1.4)	0.9 (0.7–1.3)	1.4 (1.0–2.1)
CC	33 (4.3)	26 (4.1)	0.9 (0.5–1.5)	0.9 (0.5–2.0)	0.8 (0.3–1.9)
AC/CC	263 (34.5)	222 (34.8)	1.1 (0.8–1.3)	0.9 (0.7–1.3)	1.3 (0.9–1.9)
*IL10* G-C alleles					
0	243 (29.6)	166 (24.1%)	Referent	Referent	Referent
1	393 (47.8)	348(50.4%)	1.3 (1.0–1.7)	1.3 (0.9–1.8)	1.3 (0.9–2.0)
2	186 (22.6)	176 (25.5%)	1.4 (1.0–1.9)	1.2 (0.8–1.7)	1.8 (1.1–3.0)
Test for trend			p = 0.031	p = 0.36	p = 0.020
*PTGS2*					
GG	32 (3.8)	29 (4.2)	Referent	Referent	Referent
AG	260 (31.1)	209 (30.2)	0.9 (0.5–1.5)	1.0 (0.5–2.0)	0.5 (0.2–1.4)
AA	544 (65.1)	454 (65.6)	0.8 (0.5–1.4)	1.1 (0.5–2.1)	0.5 (0.2–1.2)
GG/AG	292 (34.9)	238 (34.2)	Referent	Referent	Referent
AA	544 (65.1)	454 (65.6)	0.8 (0.5–1.4)	1.1 (0.6–2.1)	0.5 (0.2–1.2)
*IL4*					
CC	608 (75.9)	512 (76.0)	Referent	Referent	Referent
CT	174 (21.7)	150 (22.3)	1.1 (0.8–1.4)	1.1 (0.8–1.5)	1.1 (0.7–1.7)
TT	19 (2.4)	12 (1.8)	0.7 (0.3–1.4)	0.4 (0.1–1.0)	3.1 (0.7–13.9)
CT/TT	193 (24.1)	162 (24.0)	1.0 (0.8–1.3)	1.0 (0.7–1.3)	1.2 (0.8–1.8)
*IL4R* var alleles					
0	170 (23.1)	131 (21.1)	Referent	Referent	Referent
1–2	481 (65.4)	429 (69.0)	1.2 (0.9–1.5)	1.5 (1.1–2.1)	0.7 (0.4–1.1)
3–4	84 (11.4)	62 (10.0)	0.9 (0.6–1.4)	1.1 (0.7–1.9)	0.6 (0.3–1.3)
Test for trend			p = 0.98	p = 0.28	p = 0.11

a ORs adjusted for gender, age at tumor diagnosis, skin reaction to acute sun exposure, and lifetime number of severe sunburn.

When stratified by gender, the *IL10* association appeared to be largely restricted to women for both histologies. For BCC and SCC, women with 2 *IL10* GC haplotypes had an OR of about 2 (OR_BCC_ = 2.2, 95%CI 1.4–3.4; OR_SCC_ = 1.8, 95% CI 1.1–3.0), and these trends were significant (BCC: p = 0.0005; SCC: p = 0.020). Again, after a Bonferroni correction for 20 independent test (10 genes in men and 10 genes in women, p<0.0025), this trend remains significant in BCC in women, but not SCC under this more stringent requirement. Among men, the ORs were close to one (BCC: OR = 1.1, 95% CI 0.8–1.6; SCC: OR = 1.2, 95% CI 0.8–1.7). As previously reported [27], *HAL* AG/GG was associated with increased risk of SCC in women (Table 4, OR = 1.5, 95% CI 1.0–2.1). Finally, 1–2 variant alleles of the *IL4R* genes were associated with increased OR of SCC in men (Table 4, OR = 1.5, 95% CI 1.1–2.1) but not women.

MDR analysis for BCC found the most accurate one factor model considers lifetime number of severe sunburns (testing balance accuracy (TBA) = 0.5729, cross validation consistency (CVC) = 10/10, p<0.001), but a 3 factor model including a combination of skin type, burns, and *IL4R* genotype (TBA = 0.5684, CVC = 9/10, p<0.002) and a 4 factor model adding *IL10* haplotypes (TBA = 0.5627, CVC = 8/10, p<0.005) were also statistically significant. The best model of SCC included skin type and sunburns (TBA = 0.5794, CVC = 9/10, p<0.001). A three factor model including *IL4R* haplotypes (TBA = 0.5718, CVC = 4/10, p<0.001) was also statistically significant but not consistently computed as the best model. Of borderline statistical significance was a four factor model including *IL10* and *IL4R* haplotypes (TBA = 0.5434, CVC = 5/10, p<0.054).

**Figure 1:**
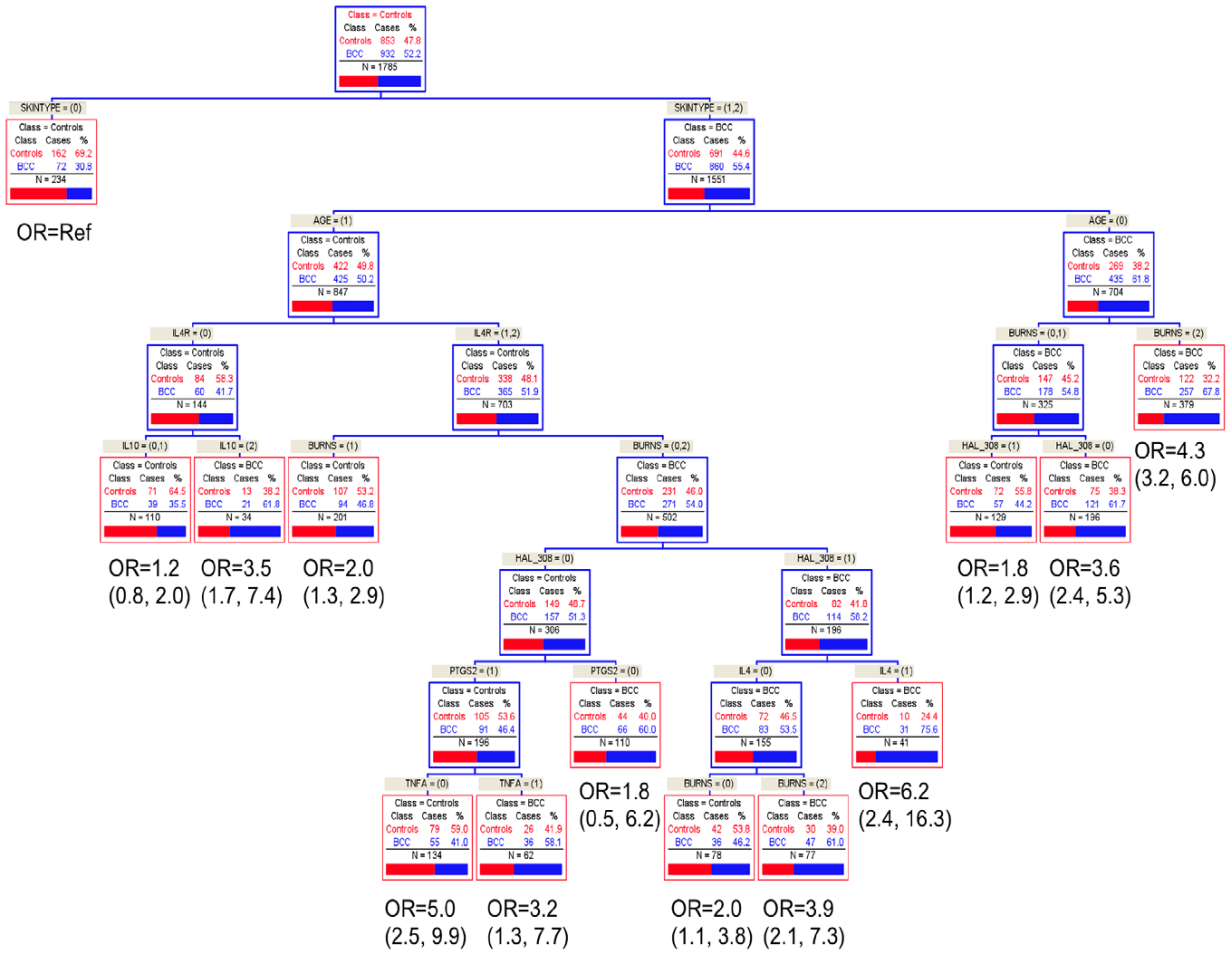
Classification and Regression Tree Analysis (CART) of BCC.

**Figure 2:**
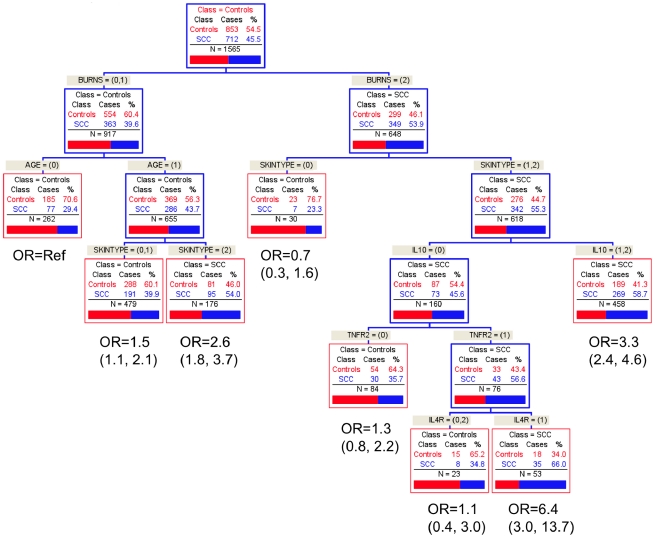
Classification and Regression Tree Analysis (CART) of SCC.

Similar results were found using CART. For BCC, combinations of *IL4R* and *IL10* and *IL4* and burns was found among those who tended to either burn or burn then tan and were older than 60 years (Figure 1). For SCC, a combination of *IL10, TNFR2,* and *IL4R* were important genetic factors among those with a high lifetime number of sunburns who tend to burn or burn then tan (Figure 2).

When examining these factors in men only, the two analysis methods identified skin type, burns, *IL10*, *IL4R*, and to a lesser extent *TNFR2* as contributing to risk of BCC. In men, the best models in MDR consider lifetime number of severe sunburns (TBA = 0.5776, CVC = 10/10, p<0.002) and a combination of skin type, burns, and *IL10* and *IL4R* haplotypes (TBA = 0.5764, CVC = 10/10, p = 0.002). Similarly, analysis using CART found *IL4R, IL10*, and *TNFR2* best predicted risk among those who tended to burn or burn then tan and were older than 60 years.

In women only, both analysis methods found combinations of skin type, burns, *IL10*, *HTR2A*, *IL12B*, and *IL4R* contributed to risk of BCC. The best fit models for MDR included skin type and *IL10* (TBA = 0.5908, CVC = 10/10, p<0.008) and skin type, burns and *IL10* (TBA = 0.5998, CVC = 10/10, p<0.004). Skin type, burns, *IL10*, *HTR2A*, *IL12B*, and *IL4R* were related to BCC risk using CART.

As with BCC, the risk of SCC in men identified skin type, burns, *IL10*, *IL4R*, and possibly *TNFR2* as significant factors. In MDR, the best model involved burns and *IL10* and *IL4R* haplotypes (TBA = 0.5704, CVC = 5/10, p<0.03). Using CART, two risk groups were identified: one with burns, skin type, *IL10,* and *TNFR2*, and a second with burns, age, skin type, and *IL4R*.

Among women, both methods found skin type, burns, and *IL10* related to risk of SCC. By MDR, significant models included just skin type (TBA = 0.5863, CVC = 8/10, p<0.05) and skin type, burns, and *IL10* haplotypes (TBA = 0.5861, CVC = 8/10, p<0.05). In CART, skin type, burns, *IL10* and *TNF* best explained the variation in the data.

## Discussion

We have investigated the effects of multiple functional variants in genes of the UV-induced immunosuppression pathway in non-melanoma skin cancer etiology. We found a main effect for *IL10* haplotypes in both BCC and SCC. Stratifying on gender suggested this was primarily attributable to the strong association in women. In men, skin type, burns, *IL10*, *IL4R*, and possibly *TNFR2* were associated with both BCC and SCC. In women, skin type, burns, and *IL10* were related to risk of SCC and BCC, and risk of BCC additionally included *HTR2A*, *IL12B* and *IL4R*.

A prior study had examined the role of *IL10* polymorphisms in UV-induced immune suppression. In this phenotype study, a CA-repeat polymorphism in the promoter region was not associated with susceptibility to UV-induced immune suppression [31]. It is possible that the CA repeat does not capture the reduced expression phenotype associated with the 2 SNP haplotype in our study. Additionally, a small population size (N = 42) and the large number of CA repeat alleles could have resulted in a false negative finding. This study also examined the role of *TNF* -308 and found no association, consistent with our results.

Previous epidemiologic studies have found a similar link between *IL10* promoter polymorphisms and risk of NMSCs in immunocompromised populations. Among renal transplant patients, a higher number of GC (or GCC) alleles was found in SCC patients compared to unaffected patients [32], and secreted IL-10 levels were also higher in this group. This was not true for BCC. A study in non-transplant patients examined the association of *IL10* -1082 and BCC and also found no association [33]. Therefore, our association between BCC and *IL10* appears to be a novel finding that warrants replication in another population.

Other previous work focused on patients with epidermodysplasia verruciformis (EV), a disease characterized by defects in cell-mediated immunity that result in persistent infection with beta-type human papillomavirus (β-HPV) and increased development of non-melanoma skin cancer [34], [35]. EV patients (n = 19) were more likely to have the low expressing (no GCC) alleles than controls (n = 25), and within EV patients, those that developed malignancies (n = 14) were also more likely to have low expressing *IL10* alleles. This could indicate a different causal pathway for HPV-related NMSC that is unrelated to UV-induced immunosuppression.

A small study conducted in a Japanese population (32 BCC, 24 SCC, 50 controls) also found an inverse association between number of GC(C) alleles and risk of *in situ* SCC (Bowen's disease) in non-transplant patients [36], but no association with BCC or SCC. Bowen's disease patients are excluded from our population; therefore, we have no ability to asses this relationship. Previous studies have shown much lower prevalence of these polymorphisms in Japanese populations [37], [38], [39] compared to Caucasians. Thus, aside from the small study size ethnic variation may account for differences in our results. Further, the control group consisted partially of patients with fungal or bacterial infections, which could potentially have been related to a pre-existing immune problem.

MDR and CART analyses identified *IL10* and *IL4R* haplotypes as being related to both BCC and SCC. *IL4* has been shown to induce *IL10* production in this pathway [40]. Further, *IL4R* is necessary to up-regulate *IL10*, a key component in biasing immune reactions towards the Th2 phenotype [41].

We have analyzed the role of gender differences in genetic susceptibility to UV-induced immunosuppression. In addition to the increased susceptibility to UV-induced immunosuppression seen in the female NZB mice, evidence exists for a role of estrogen in regulating wider immune signaling. Experimental evidence supports the fact that estrogen promotes a Th-2 bias, similar to UVR. In pregnancy, when circulating estrogen levels are high, the fetus spontaneously releases Th-2 cytokines (i.e. IL-4 and IL-10), hence biasing the mother's immune system away from producing Th-1 type cytokines which are implicated in spontaneous abortions [42]. Similar to UVR, contraceptive level doses of β-estradiol result in suppression of delayed-type hypersensitivity (DTH) reactions [43]. β-estradiol inhibits the recruitment of inflammatory cells types, such as granulocytes, macrophages, and T cells, to sites of inflammation [44]. Also, T cell activation does not occur, due to improper antigen presentation from spleen antigen presenting cells (APCs) [43], [45]. Considering women may be more primed for immune suppression given the effects of β-estradiol, it is not surprising that our genetic effects were stronger in women.

One advantage of a pathway-based analysis is the ability to examine how combinations of genotypes contribute to disease risk. Often this is done by examining all of the common variants in each gene to guarantee complete coverage of all the variation in the pathway. However, this approach is costly, and the large number of data points and comparisons can increase the chances of type 2 errors. Instead, we have taken a phenotypic approach to pathway analysis, using almost exclusively SNPs that have been reported as functional in past studies (Table 1). While this does not guarantee full coverage of the pathway variation, it has the advantage of improving statistical power and improving interpretability, as the genotypes have an identified phenotype. However, it is important to note that functionality of a SNP does not guarantee that it is the causal SNP, in relation to the outcome of interest; the possibility of the causal SNP being in linkage disequilibrium with the SNP examined still remains.

Major strengths of this study include the population-based nature of the study and the large sample size included, yielding increased generalizability and power to detect smaller genetic effects. Possible limitations include recall bias and missing genetic information for all variants in the population. Sun exposure risk factors of interest in this study were determined by self-reported interview responses, which are subject to recall bias. However, standardized interview instruments like the one used in the NHHS tend to yield reproducible results upon retest for many sun exposure variables [46], [47]. Additionally, there was no evidence of differential recall of sunburns between cases and controls in either study. Finally, use of sunburns as a categorical variable instead of a continuous variable has been shown to improve reproducibility of recall [47].

While genotyping is not complete for any of the genes studied, overall the rate of missing data is relatively small. For 8 of the 10 genes examined, 94% or greater of the 2,493 participants in this study could be assigned a genotype or haplotye. *IL12B* and *IL4R* had slightly lower overall genotyping rates, at 90.1% and 87.1%, respectively. However, missing genetic information was not associated with either outcome of interest for any of the genes examined, so we do not believe that this is contributing to a substantial bias in the study.

In this study, we have examined the role of functional polymorphisms in the UV-induced immunosuppression pathway and how they alter risk for NMSC. Like many other cancers, BCC and SCC are both complex diseases where genetic predisposition and environmental exposures, as well and their interactions, contribute enormously to disease etiology. The combinations of gene and environmental (sunburn) effects observed in this study underline the importance of examining genetic variants as part of the greater pathway to which they belong. While the highly antigenic nature of NMSCs make them well-suited to examine the role of genetic variants in the UV-induced immunosuppression pathway, tumor surveillance plays a part in many types of cancers, and thus the interactions found in this study may have applications to a variety of solid tumor types.
